# NADP-Dependent Isocitrate Dehydrogenase from *Arabidopsis* Roots Contributes in the Mechanism of Defence against the Nitro-Oxidative Stress Induced by Salinity

**DOI:** 10.1100/2012/694740

**Published:** 2012-05-02

**Authors:** Marina Leterrier, Juan B. Barroso, Raquel Valderrama, José M. Palma, Francisco J. Corpas

**Affiliations:** ^1^Departamento de Bioquímica, Biología Celular y Molecular de Plantas, Estación Experimental del Zaidín, CSIC, Apartado 419, 18080 Granada, Spain; ^2^Grupo de Señalización Molecular y Sistemas Antioxidantes en Plantas, Unidad Asociada al CSIC (EEZ), Departamento de Bioquímica y Biología Molecular, Universidad de Jaén, 23071 Jaén, Spain

## Abstract

NADPH regeneration appears to be essential in the mechanism of plant defence against oxidative stress. Plants contain several NADPH-generating dehydrogenases including isocitrate dehydrogenase (NADP-ICDH), glucose-6-phosphate dehydrogenase (G6PDH), 6-phosphogluconate dehydrogenase (6PGDH), and malic enzyme (ME). In *Arabidopsis* seedlings grown under salinity conditions (100 mM NaCl) the analysis of physiological parameters, antioxidant enzymes (catalase and superoxide dismutase) and content of superoxide radical (O_2_^  ∙−^), nitric oxide (NO), and peroxynitrite (ONOO^−^) indicates a process of nitro-oxidative stress induced by NaCl. Among the analysed NADPH-generating dehydrogenases under salinity conditions, the NADP-ICDH showed the maximum activity mainly attributable to the root NADP-ICDH. Thus, these data provide new insights on the relevance of the NADP-ICDH which could be considered as a second barrier in the mechanism of response against the nitro-oxidative stress generated by salinity.

## 1. Introduction

In higher plants, salinity can provoke alterations in the metabolism of proteins and nucleic acids, photosynthesis and respiration [1–3]. In addition, the production and participation of reactive oxygen species (ROS) during different plant stress conditions including salinity is also well documented [4–8], and more recently the involvement of nitric oxide (NO) and related molecules designated as reactive nitrogen species (RNS) seems to be also a complementary part of the mechanism of response of plants against environmental stresses [9] which can participate in a nitro-oxidative stress situation.

NADPH is a key cofactor in the cellular redox homeostasis, being an indispensable electron donor in numerous enzymatic reactions, biosynthetic pathways, and detoxification processes [10, 11]. In this sense, NADPH is necessary in the metabolism of ROS and RNS; for example, it is a reducing equivalent for the regeneration of reduced glutathione (GSH) by glutathione reductase (component of ascorbate-glutathione cycle) and for the activity of the NADPH-dependent thioredoxin system, two important cell antioxidants against oxidative damage. Moreover, NADPH is also required for the generation of superoxide radical by the NADPH oxidase (NOX) [12], but is also a necessary cofactor for the generation of nitric oxide (NO) by the L-arginine-dependent nitric oxide synthase activity [13]. The most important enzymes which have the capacity to generate reducing power in the form of NADPH in plants are the ferredoxin-NADP reductase as a component of photosystem I [14] and a group of NADP-dehydrogenases located in different subcellular compartments which includes the NADP-isocitrate dehydrogenase (NADP-ICDH), the glucose-6-phosphate dehydrogenase (G6PDH) and 6-phosphogluconate dehydrogenase (6PGDH) (both belonging to the pentose phosphate pathway), and the NADP-malic enzyme (ME) [15–17]. Among the different NADP-ICDH isoforms present in higher plants, it has been shown that the cytosolic NADP-ICDH represents more than 90% of the total cellular NADP-ICDH activity [18–21], and very recently *in vitro* assays have shown that the *Arabidopsis* cytosolic NADP-ICDH activity from *Arabidopsis* roots and leaves is differentially regulated by molecules involved in ROS and RNS metabolism [22] including H_2_O_2_, NO, and ONOO^−^ indicating a metabolic interconnection among this enzyme and these molecules.

In the present work, using *Arabidopsis* as model plant, it is shown that under salinity (100 mM NaCl) stress there is a concomitant nitro-oxidative imbalance that is accompanied by a general induction of NADP-dehydrogenase activities being the NADP-ICDH from roots, the enzyme with the most prominent activity. The present data support that the recycling of NADPH is important as a mechanism against cellular nitro-oxidative damage produced by salinity.

## 2. Material and Methods

### 2.1. Plant Material and Growth Conditions

*Arabidopsis thaliana* ecotype Columbia seeds were surface sterilized for 5 min in 70% (v/v) ethanol containing 0.1% (w/v) SDS, then placed for 20 min in sterile water containing 20% (v/v) bleach and 0.1% (w/v) SDS, and washed four times in sterile water. The seeds were sown for 2 days at 4°C in the dark for vernalization on the basal growth medium composed of 4.32 g/L commercial Murashige and Skoog medium (Sigma) with a pH of 5.5, containing 1% (w/v) sucrose and 0.8% (w/v) phyto agar. The Petri plates containing the *Arabidopsis* seeds were then grown at 22°C/18°C (16 h light/8 h dark, long-day conditions) under a light intensity of 100 *μ*E m^−2^ s^−1^. For the experiments with NaCl stress, 6-day-old seedlings were transferred to MS medium plates both with and without 100 mM NaCl for another 7 days under long-day conditions [23].

### 2.2. Crude Extracts of Plant Tissues

*Arabidopsis* seedlings were frozen in liquid N_2_ and ground in a mortar with a pestle. The powder was suspended in a homogenizing medium containing 50 mM Tris-HCl, pH 7.8, 0.1 mM EDTA, 0.2% (v/v) Triton X-100, and 10% (v/v) glycerol. Homogenates were centrifuged at 27,000 g for 20 min, and the supernatants were used for the assays.

### 2.3. Histochemical Analyses

Histochemical detection of plasma membrane loss integrity in *Arabidopsis* root apexes was performed by the method described by Yamamoto et al. [24]. For this analysis, the *Arabidopsis* seedlings were incubated in 15 mL of Evans blue solution [0.2% (w/v) in water] for 10 min, and then they were washed three times in distilled water for 10 min each. Blue color indicates damage to the plasma membrane.

### 2.4. Enzymatic Activity Assays

Catalase activity (EC 1.11.1.6) was determined by measuring the disappearance of H_2_O_2_, as described by Aebi [25]. Glycolate oxidase (GOX; EC 1.1.3.1) was assayed as described previously [26] by measuring the formation of glyoxylate-phenylhydrazone. Hydroxypyruvate reductase (HPR) was assayed according to Schwitzguébel and Siegenthaler [27].

Glucose-6-phosphate dehydrogenase (G6PDH; EC 1.1.1.49) activity was determined spectrophotometrically by recording the reduction of NADP at 340 nm. Assays were performed at 25°C in a reaction medium (1 mL) containing 50 mM HEPES, pH 7.6, 2 mM MgCl_2_, and 0.8 mM NADP, and the reaction was initiated by the addition of 5 mM glucose-6-phosphate. For the determination of 6-phosphogluconate dehydrogenase (6PGDH; EC 1.1.1.44) activity, the reaction mixture was similar to that described for G6PDH, but the substrate was 5 mM 6-phosphogluconate [28]. NADP-isocitrate dehydrogenase (NADP-ICDH, EC 1.1.1.42) activity was also measured by following the NADP reduction according to Corpas et al. [29]. Thus, the assay was performed at 25°C in a reaction medium (1 mL) containing 50 mM HEPES, pH 7.6, 2 mM MgCl_2_ and 0.8 mM NADP, and the reaction was initiated by the addition of 10 mM 2R,3S-isocitrate. NADP-malic enzyme (NADP-ME; EC 1.1.1.40) activity was also determined spectrophotometrically by recording the reduction of NADP at 340 nm using the same reaction mixture (1 mL) indicated above for other dehydrogenases, but in this case, the reaction was initiated by the addition of 1 mM L-malate [30].

### 2.5. Superoxide Dismutase Isozymes

Superoxide dismutase (SOD; EC 1.15.1.1) isozymes were separated by native PAGE on 12% acrylamide gels and visualized by a photochemical NBT (nitroblue tetrazolium) reduction method [31]. To identify the type of SOD isozymes, gels were preincubated separately at 25°C for 30–45 min in 50 mM K-phosphate, pH 7.8, in the presence or absence of either 2 mM KCN or 5 mM H_2_O_2_. CuZn-SODs are inhibited by CN^−^ and H_2_O_2_, Fe-SODs are inhibited by H_2_O_2_ but not by CN^−^, whilst Mn-SODs are not inhibited by either CN^−^ or H_2_O_2_ [32].

### 2.6. RNA Isolation and Semiquantitative RT-PCR

Total RNA was extracted with Trizol according to Gibco BRL, Life Technologies. Two *μ*g of total RNA were used to produce cDNA by RT-PCR. Semiquantitative reverse transcription-PCR amplification of actin cDNA from *Arabidopsis* was chosen as control. *NADP-ICDH* and *actin* cDNAs were amplified by the PCR as follows: 1 *μ*L of each cDNA (30 ng) was added to 250 mM dNTPs, 1.5 mM MgCl_2_, 1 × PCR buffer, 0.5 U of Hot Master TaqTM DNA polymerase (Eppendorf), and 0.5 mM of each primer (cytosolic *ICDH*: 5′-TTGTGGAGAGGAGTGTTGAG-3′ and 5′-CCTAAAAGACCCTAATACCA-3′; mitochondrial/chloroplastic *ICDH* 5′-GGGAATTGGGAACAATACA-3′ and 5′-TGTTGGATACGAAACTGAA-3′; peroxisomal *ICDH*: 5′-CAGCGTGATGTTTGATTTG-3′ and 5′-TAGCCATTTCTGTTGATTGG-3′; *actin II*: 5′-TCCCTCAGCACATTCCAGCAGAT-3′ and 5′-AACGATTCCTGGACCTGCCTCATC-3′) in a final volume of 20 *μ*L. Reactions were carried out in a Hybaid thermocycler. A first step of 2 min at 95°C was followed by 28 cycles of 20 s at 94°C, 20 s at 55°C, and 30 s at 65°C plus a final step of 10 min at 65°C. Then, PCR products were detected by electrophoresis in 1% (w/v) agarose gels and staining with ethidium bromide. Quantification of the bands was performed using a Gel Doc system (Bio-Rad Laboratories) coupled with a high-sensitive charge-coupled device (CCD) camera.

### 2.7. Detection of Superoxide Radical (O_2_^∙−^), Nitric Oxide (NO), and Peroxynitrite (ONOO^−^) by Confocal Laser Scanning Microscopy (CLSM)

Detection of superoxide radicals (O_2_^∙−^) in roots of *Arabidopsis* seedlings was carried out using 10 *μ*M dihydroethidium (DHE) [33] by incubation of *Arabidopsis* seedlings with this fluorescent probe for 1 h at 37°C in darkness.

Nitric oxide (NO) and peroxynitrite (ONOO^−^) were detected using the fluorescent reagents 10 *μ*M of 4-aminomethyl-2′,7′-difluorofluorescein diacetate (DAF-FM DA, Calbiochem) and 10 *μ*M 3′-(*p*-aminophenyl) fluorescein (APF, Invitrogen), respectively, according to Corpas et al. [34].

In all cases, the images obtained by CLSM system (Leica TCS SL; Leica Microsystems, Wetzlar, Germany) from control and treated *Arabidopsis* seedlings were maintained constant during the course of the experiments in order to produce comparable data. The images were processed and analyzed using statistical Leica-Lite software.

### 2.8. Other Assays

Protein concentration was determined with the Bio-Rad Protein Assay (Hercules, CA) using bovine serum albumin as standard. To estimate the statistical significance between means, the data was analyzed by Student's *t* test.

## 3. Results

### 3.1. Effect of Salinity in Physiological Parameters and in the Metabolism of Reactive Oxygen Species (ROS)

**Figure 1:**
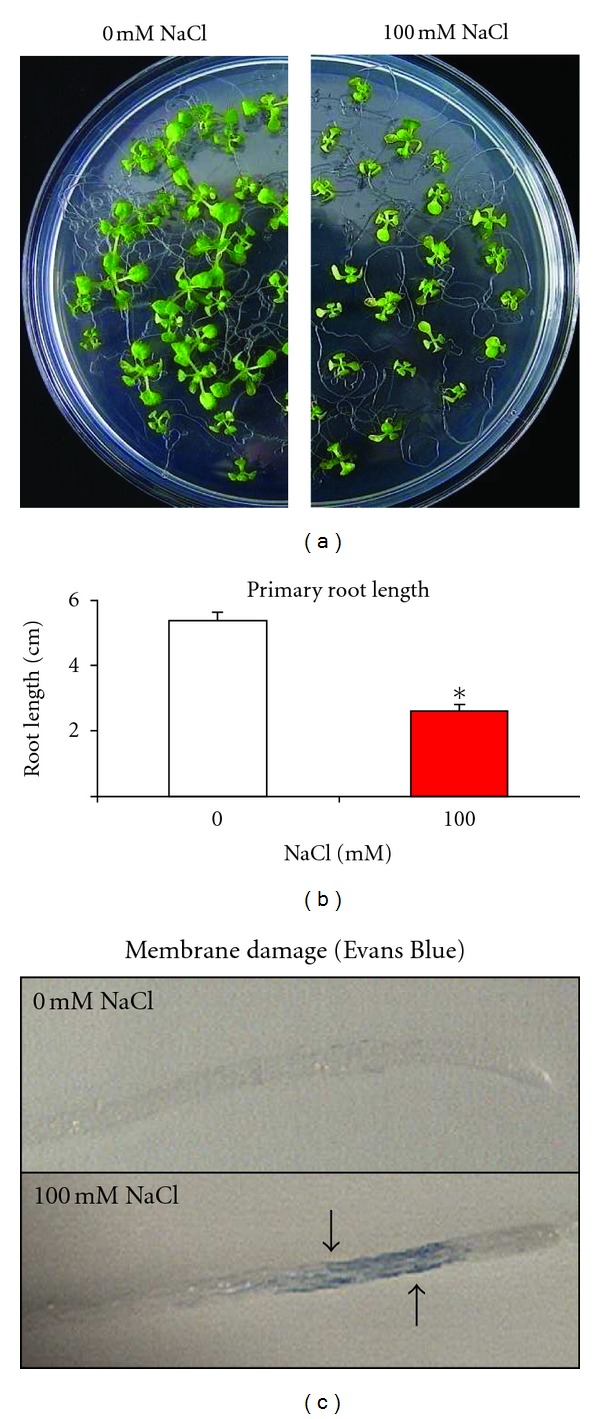
Effect of salinity in *Arabidopsis* seedlings growth. (a) Appearance of 14-day-old *Arabidopsis* seedling growth in MS medium supplemented and nonsupplemented with 100 mM NaCl. (b) Primary root length. Results are the mean of three different experiments ± SEM. *Differences in relation to control values were significant at *P* < 0.05. (c) Histochemical detection of plasma membrane integrity by staining with Evan blue solutions. Blue colour (arrows) indicates the root area where the membrane integrity is affected by salinity.

Previous studies have shown that *Arabidopsis* seedlings grown with 100 mM NaCl underwent salinity stress [23, 35], and therefore this concentration was chosen for the salinity treatment.  Figure 1(a) shows the appearance of *Arabidopsis* seedlings grown with 100 mM NaCl. These seedlings had a smaller size, leaves with chlorotic symptoms, and a root length reduced by 24% (Figure 1(b)). To determine whether NaCl could affect the cell-membrane integrity of the root cells, a histochemical method based on the Evans Blue staining was used. Thus, an intense blue color appeared in roots of seedlings exposed to 100 mM NaCl, indicating the loss of cell-membrane integrity (Figure 1(c)).

**Figure 2:**
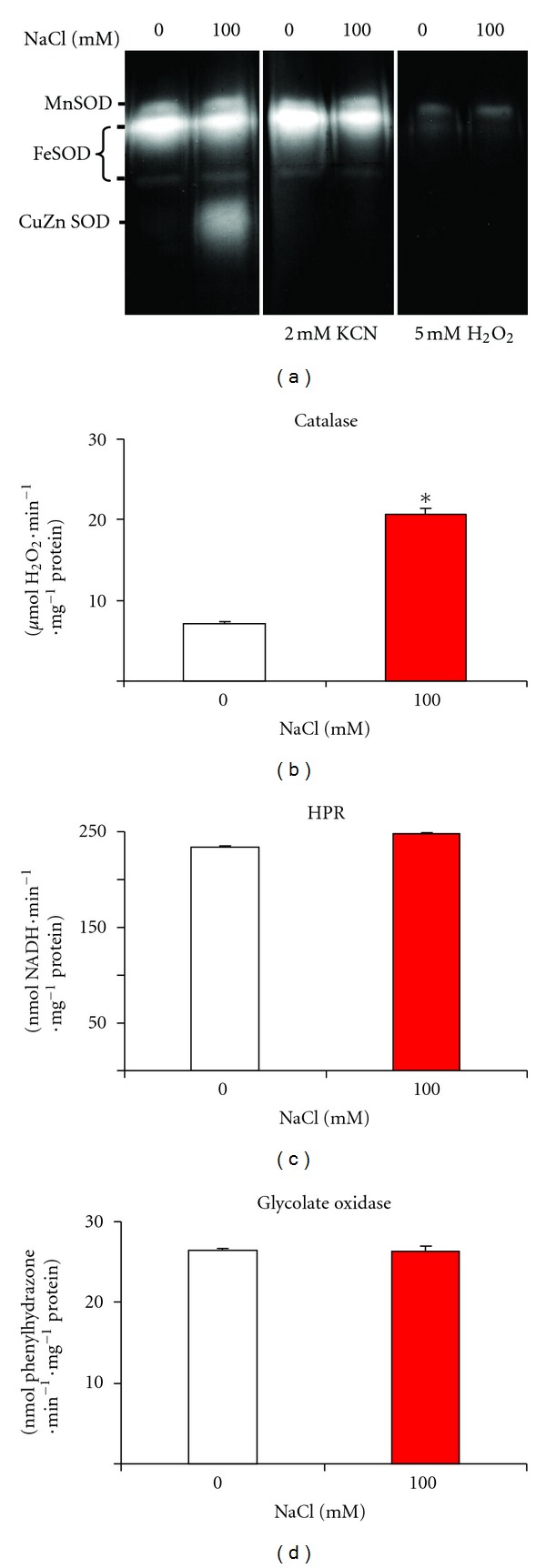
Enzyme activities in *Arabidopsis* seedlings exposed to 100 mM NaCl. (a) Superoxide dismutase (SOD) isoenzymes activities in native gels incubated in the absence and the presence of specific inhibitors, either cyanide or hydrogen peroxide. (b) Catalase activity. (c) Hydroxypruvate reductase (HPR) activity. (d) Glycolate oxidase activity. Results are the mean of three different experiments ± SEM. *Differences in relation to control values were significant at *P* < 0.05.

To establish whether our experimental salinity conditions affect ROS metabolism, the activity of the first line of antioxidant enzymes was analyzed, including superoxide dismutase (SOD) and catalase, and some key enzymes of the photorespiratory pathway (NADH-hydroxypyruvate reductase and glycolate oxidase). The analysis of SOD activity in native gel showed the presence of four isozymes which differed according to their susceptibility to the inhibitor, whether cyanide or hydrogen peroxide: one Mn-SOD, two Fe-SODs, and one CuZn-SOD, which displayed increasing electrophoretic mobility (Figure 2). As can also be seen, only the CuZn-SOD isozyme was strongly induced by salinity without significantly affecting the other SOD isozymes (Figure 2(a)). On the other hand, the catalase activity increased 2.9-fold under salinity conditions (Figure 2(b)). However, the hydroxypyruvate reductase (HPR) and the glycolate oxidase activities were not affected after 100 mM NaCl treatment (Figures 2(c) and 2(d), resp.).

### 3.2. Cellular Analysis of Superoxide Radical (O_2_^∙−^), NO, and ONOO^−^ Production Induced by Salinity

**Figure 3:**
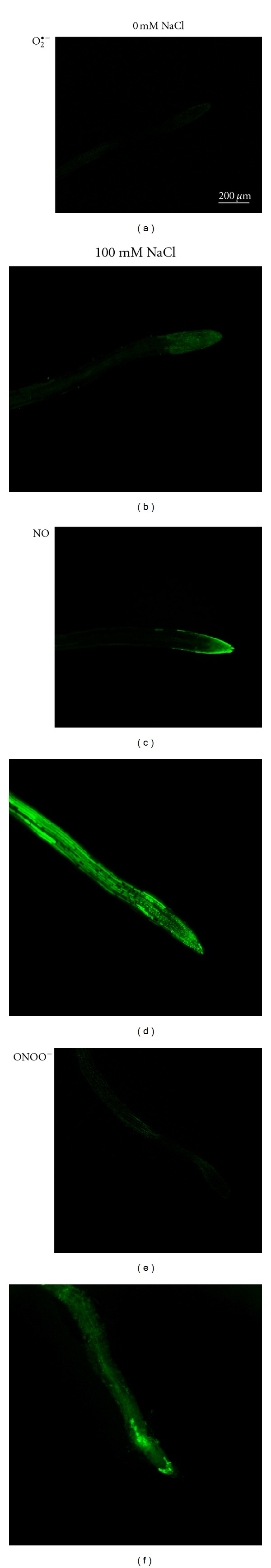
Representative images illustrating the CLSM* in vivo* detection of superoxide radical (O_2_^∙−^) ((a) and (b)), nitric oxide (NO) ((c) and (d)), and peroxynitrite (ONOO^−^) ((e) and (f)) in root tips of *Arabidopsis* seedlings exposed to 100 mM NaCl.

Figure 3(a) to 3(f) shows the analysis by confocal laser scanning microscope (CLSM) of the content of O_2_^∙−^, NO, and ONOO^−^ in the root tips of *Arabidopsis* seedlings exposed to 100 mM NaCl. The cellular production of O_2_^∙−^ was analyzed using the fluorescent probe DHE, which is specific for this radical. In control seedlings, the green fluorescence corresponding to O_2_^∙−^ was slightly detected in the root tips (Figure 3(a)). However, in roots from NaCl-treated seedlings, the green fluorescence was intensified in the root tips (Figure 3(b)). When NO generation was analyzed using DAF-FM DA as the fluorescence probe, a significant increase in NO production (green color) was noted in the roots under salt stress with a homogenous distribution throughout the root (Figures 3(c) and 3(d)), whereas in control plants, labeling was detected only in root tips. On the other hand,  ONOO^−^, which results from the reaction between O_2_^∙−^ and NO was also analyzed in roots by CLSM using the fluorescence probe APF.  Figure 3(e) shows the location of ONOO^−^ in the control roots of *Arabidopsis* seedlings with very slight fluorescent signal. However, this RNS significantly increased in roots under salinity stress with a homogeneous distribution throughout the root (Figure 3(f)), similar to the distribution of the NO.

### 3.3. Effect of Salinity on NADP-Dehydrogenase Activities

**Figure 4:**
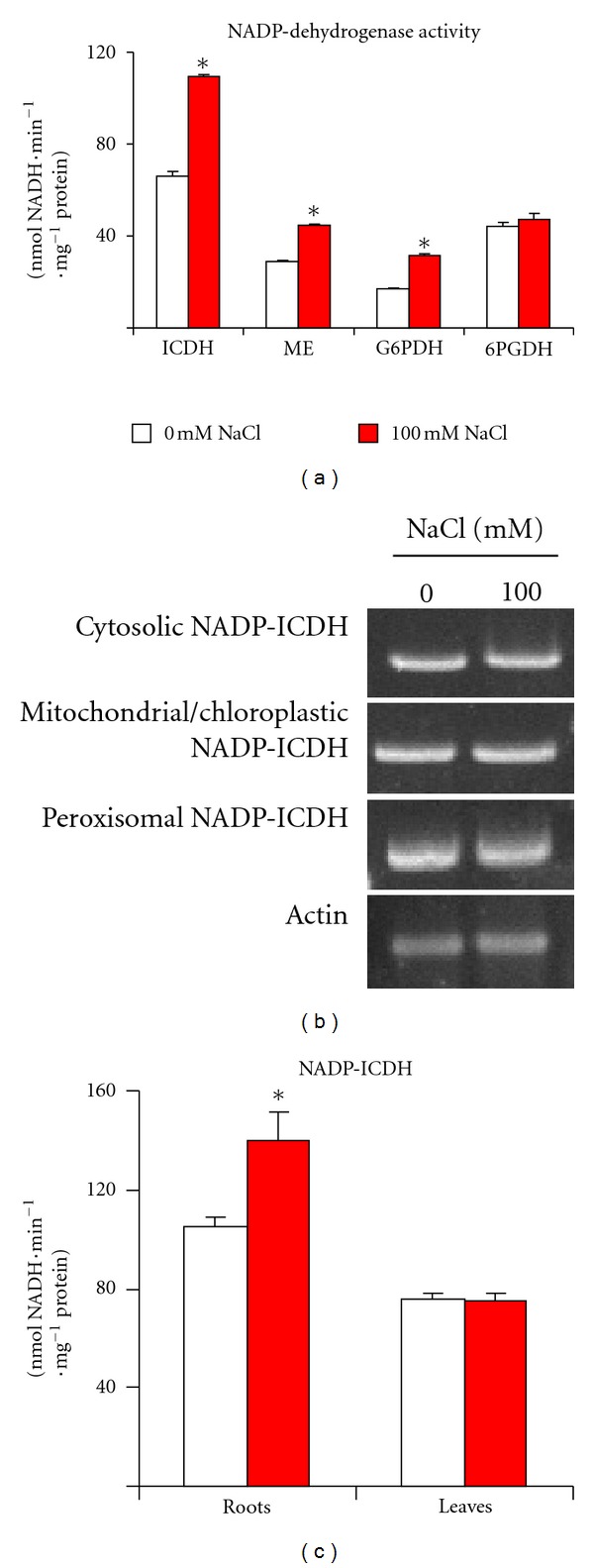
(a) Activity of NADP-isocitrate dehydorgenase (ICDH), malic enzymes (ME), glucose-6-phosphate dehydrogenase and 6-phosphogluconate dehydrogenase in *Arabidopsis* seedlings exposed to 100 mM NaCl. (b) Representative agarose electrophoresis gel of the semiquantitative RT-PCR analysis of the cytosolic (At1g65930), mitochondrial/chloroplastic (At5g14590), and peroxisomal (At1g54340) *NADP-ICDH* genes in *Arabidopsis* seedlings exposed to 100 mM NaCl. Gel was visualized by ethidium bromide staining, and *actin *was used as internal control. (c) NADP-ICDH activity in roots and leaves of *Arabidopsis* seedlings exposed to 100 mM NaCl. Results are the mean of three different experiments ± SEM. *Differences in relation to control values were significant at *P* < 0.05.

The analysis of the activity of the main NADP dehydrogenases is shown in Figure 4(a). The activity of NADP-ICDH, ME, and G6PDH increased 1.6-, 1.5-, and 1.9-fold, respectively, with respect to control seedlings. However, the 6PGDH activity was not affected by salinity treatment. Considering that the NADP-ICDH showed a higher relative specific activity under salinity conditions in comparison to the other NADP dehydrogenases, further analyses were focused on this enzyme.

*Arabidopsis* has several NADP-ICDH isoforms localized in different subcellular compartments including the cytosol, chloroplasts/mitochondria and peroxisomes [20]. For an evaluation of the potential contribution of each isoform under salinity stress, its gene expression was analyzed.  Figure 4(b) showed the gene expression of the cytosolic (At1g65930), chloroplastic/mitochondrial (At5g14590) and peroxisomal (At1g54340) NADP-ICDH evaluated by semiquantitative RT-PCR. Contrary to what happened in the activity analysis, none of the genes appeared to undergo significant changes under salinity stress. With the goal of gaining fuller knowledge of the potential function of the NADP-ICDH activity, its activity was investigated independently in roots and leaves of *Arabidopsis* seedlings after 100 mM NaCl treatment (Figure 4(c)). NADP-ICDH activity was found to be higher in roots than in leaves from control plants. Also, it was observed that, under salinity conditions, the activity significantly increased (by 39%) in roots whereas the activity in leaves showed no change.

## 4. Discussion

Salinity is recognized to influence plant productivity due to its negative effects on plant growth, ion balance, and water relations. In addition, in many plant species such as pea [4, 5], tomato [36, 37], or olive [30], the salinity stress is usually accompanied by an oxidative stress. In this sense, the data gathered in our *Arabidopsis in vitro* model system corroborate that salinity (100 mM NaCl) significantly reduces root growth, damages root plasma-membrane integrity, boosts the production of superoxide radical, and significantly raises catalase and CuZn-SOD activities, although photorespiration appears not to be affected. The remarkable induction of a CuZn-SOD in salt-treated *Arabidopsis* seedlings, enhances the relevance of this enzymatic system in the response of plants to salinity stress, as has been found earlier [5, 30, 36]. On the other hand, the analysis of some RNS such as nitric oxide (NO) and peroxynitrite (ONOO^−^) also showed a higher content under salinity stress, which also agrees with previous data in different plant species [33, 34, 38, 39]. Therefore, in this context, where the ROS and RNS metabolism is affected under salinity stress, the analysis of NADPH-generating dehydrogenase activity was studied, considering that NADPH is necessary for the metabolism of these species because it occurs in some antioxidant systems such as the ascorbate-glutathione cycle, the generation of superoxide radical (O_2_^∙−^) by the NADPH oxidase [12], and NO generation by a L-arginine nitric oxide synthase [13, 14]. Thus, the general increase in the activity of these NADP-dehydrogenases is reasonable considering the increase of peroxynitrite observed in roots. This molecule, being a strong oxidant which results from the interaction of (O_2_^∙−^) and NO, must provoke cellular damage. Consequently, the general increase of the NADPH-generating dehydrogenases, with the exception of the 6PGDH, suggests the participation of these enzymes in the mechanism of response against the nitro-oxidative stress prompted by the salinity treatment. Accordingly, in dune reed (*Phragmites communis*) callus under 50–150 mM NaCl treatments, the G6PDH activity was induced, being necessary for GSH maintenance and H_2_O_2_ accumulation under salt stress [40]. Furthermore, in *Carex moorcroftii *callus under salt stress (100 mM NaCl), G6PDH was also involved in the regulation of plasma membrane H^+^-ATPase [41]. These results also agree with the behavior of these NADP dehydrogenases under other kinds of environmental stress such as cadmium [42] or low temperature [43] where the activity of some of these NADP-dehydrogenases was induced.

Among these NADP dehydrogenases, special attention was placed on NADP-ICDH, since this activity was higher than that of other NADPH-generating dehydrogenases. In previous works, it has been reported that the NADP-ICDH was significantly greater in oxidative stress situation promoted after paraquat treatment in pea nodule [44], biotic stress in *Arabidopsis* [21], mechanical wounding, high and low temperature in pea leaves [26], and low temperature in pepper leaves [43], thus indicating the contribution of NADP-ICDH to the redox state of the cell. In the facultative halophyte *Mesembryanthemum crystallinum* adapted to high salinity (400 mM), the NADP-ICDH activity increased in leaves and decreased in roots [45]. However, in our experimental model of *Arabidopsis*, the comparison of NADP-ICDH activity between the two organs (roots and leaves) points to a significant role of this enzyme in roots. This difference in NADP-ICDH activity between the two organs in *M. crystallinum* and *A. thaliana* must be related to the degree of resistance to salinity in each plant species, and thus the increase of the NADP-ICDH activity must be related to the NADPH requirement in each organ. Thus, in the case of *M. crystallinum* under salt stress the excess Na^+^ is transported very efficiently to the leaves whereas only a minor part is accumulated in root tissue [46]; however, in *Arabidopsis* the situations is totally different, considering the sensitivity of this plant to salinity in comparison to *M. crystallinum* [2].

In summary, these data suggest that the activities of the NADPH-generating dehydrogenases, especially the NADP-ICDH in roots, contributed to maintaining the cellular redox status as a mechanism to support the antioxidative system during the nitro-oxidative stress generated by salinity stress in *Arabidopsis*. Thus, it is proposed that NADP-ICDH dehydrogenase acts in *Arabidopsis* seedlings as a second barrier in the response mechanism of salinity stress, but they could also have a protective function in other types of abiotic stress.
